# Gut Microbiome Composition Associated With Major Depressive Disorder and Sleep Quality

**DOI:** 10.3389/fpsyt.2021.645045

**Published:** 2021-05-21

**Authors:** Qi Zhang, Yajun Yun, Huimei An, Wenxuan Zhao, Ting Ma, Zhiren Wang, Fude Yang

**Affiliations:** ^1^Peking University HuiLongGuan Clinical Medical School, Beijing HuiLongGuan Hospital, Beijing, China; ^2^College of Basic Medical and Clinical Pharmacy, China Pharmaceutical University, Nanjing, China

**Keywords:** gut microbiome, major depressive disorder, sleep quality, 16S rRNA sequencing, insomnia

## Abstract

The microbiota–gut–brain axis plays a critical role in the pathogenesis of major depressive disorder (MDD) and related subclinical symptoms. However, studies on the gut microbiota in MDD are inconsistent, and data on MDD's effects on sleep are lacking. This study aimed to analyze the gut microbiota composition and sleep quality of patients with MDD. We performed 16S rRNA sequencing of stool samples from 36 patients with MDD and 45 healthy controls (HC). Sleep quality was assessed using the Pittsburgh Sleep Quality Index, depressive severity with the Hamilton Depression Scale, and insomnia severity using the Insomnia Severity Index. Forty-eight microbiota targets showed significant differences between MDD and HC. In MDD, six microbiota targets were associated with the severity of depression, 11 with sleep quality, and 3 with sleep severity. At the genus level, *Dorea* was simultaneously related to depression and sleep quality, while *Intestinibacter* was more closely related to sleep problems. *Coprococcus* and *Intestinibacter* were associated with sleep quality independent of the severity of depression. In conclusion, the present findings enable a better understanding of the relationship between gut microbiota and MDD-related symptoms. Gut microbiota alterations may become potential biomarkers and/or treatment targets for sleep quality in MDD.

## Introduction

Major depressive disorder (MDD) is a common psychiatric illness influencing ~300 million people (1), with lifetime prevalence rates of about 10.8% as reported in a survey of 30 countries (2) and exerting a huge clinical and social burden.

Several hypotheses, including the monoamine hypothesis, the subclinical inflammation hypothesis (3, 4), and hypothalamic–pituitary axis (HPA) dysregulation, have been proposed to explain its underlying etiology (5). Another promising hypothesis is gut–brain axis (GBA) dysfunction (6). The gut microbiota affects both the gastrointestinal system and central nervous system (CNS) function. Bacteria can produce neurotransmitters such as GABA, dopamine, and serotonin which affect emotional and sleep states (7). Because of their neuroactive properties and their effects on other gut–brain signaling pathways, including immune and endocrine systems, the main metabolites produced by intestinal dietary fiber bacterial fermentation, short-chain fatty acids (SCFAs), are speculated to be directly or indirectly involved in communication along the brain–gut axis (8, 9).

Several studies have attempted to prove the association between the gut microbiota and depression, but with inconsistent results. For instance, Jiang et al. found that the gut microbiome diversity in MDD was higher than that in HC, and the proportion of *Bacteroides, Proteus*, and *Actinomycetes* was significantly higher than in HC (10). Chung et al. found no significant difference in bacterial abundance and diversity between the two groups, but found increasing *Actinobacteria* and *Firmicutes* and decreasing *Bacteroidetes* and *Proteobacteria* in MDD. Furthermore, they found that the severity of depression correlated with bacterial composition (11). Aside from anhedonia and the enduring depressed mood, sleep disturbance is a common issue for MDD patients, present during the whole course of the disease even as a residual symptom. More than 90% of patients with depression have sleep disorders, and a small number of patients complain of drowsiness (12). Previous hypothesis considered that sleep and depression were a one-way causal relationship in which depression leads to sleep disorders, but new evidence seems to claim a bidirectional relationship between them. However, at present, no universally acknowledged theory explains the pathophysiologic mechanism between depression and sleep. In the past decade, known theories including the S-deficiency hypothesis (13), HPA dysfunction hypothesis (14), circadian rhythm (15, 16), rapid eye movement (REM) phase advance hypothesis (17), and neuroimmune mechanisms (18) can only partly explain the cause of insomnia in depression. Therefore, a novel mechanism involving the gut–brain axis has been proposed (12, 13, 19).

There is considerable evidence indicating that gut microbiome can regulate sleep and mental states (20). Previous studies have shown a positive association between sleep quality, the F/B ratio, a greater relative abundance of *Blautia* and *Ruminococcus* (*Firmicutes*), and lower proportions of *Prevotella, Bacteroidetes*, and *TM7-3a* (21, 22). Despite the lack of research on this topic in the context of depression, three cross-sectional studies analyzing sleep and intestinal microbiota in bipolar disorder and irritable bowel syndrome found negative correlations between *Faecalibacterium, Lactobacillus*, and sleep quality (23, 24) among bipolar disorder patients, while baseline intestinal and gut microbiota diversity had a negative correlation with the Hamilton Depression Rating Scale (HAM-D) score in irritable bowel syndrome (25). Therefore, to fill the evidence gap in MDD, the current study focused on (1) characterizing the gut microbiota distributions of participants with MDD and (2) determining whether gut bacteria differentially correlate with sleep quality.

## Methods and Materials

### Participants

We recruited 36 patients with MDD and 45 healthy controls from inpatients at Peking University Huilongguan Clinical Medical School, Beijing Huilongguan Hospital between January 2020 and October 2020. The MDD group inclusion criteria were as follows: (1) 18–55 years of age, (2) diagnosed with MDD and a depressive episode of at least moderate severity according to ICD-10 criteria (F32.1, F32.2, F33.1, F33.2) by two trained psychiatrists (26), (3) total scores of the 17-item version HAM-D >17 (26), and (4) drug naive or without treatment for ≥1 week and without long-acting antipsychotics >6 months before the study. Healthy controls were recruited from nearby communities and were screened out with any history of psychiatric disorders or psychosis among their first-degree relatives. The candidates of HC were hospital staff, care workers, and patients' accompanying family members and friends with no consanguinity. For all participants in our study, physical examination results, laboratory test results (blood and urine analyses), imaging results, and past history were collected before admission to exclude those with history of persistent infection, allergy, or inflammatory diseases whether systematic or local inflammation. Exclusion criteria for both patients and control groups were as follows: (1) a prior medical history of central nervous system disease, severe head injury, substance abuse or dependence, intellectual disability, and other severe medical records; (2) recent use of antibiotics or probiotic synbiotics within 30 days of study participation; (3) history of gastrointestinal surgery or severe congenital abnormalities; (4) night shift or rotating schedule within the past 3 months; (5) history of electroconvulsive therapy within the previous 6 months; (6) pregnancy; and (7) comorbidities associated with other sleep disorders (e.g., sleep apnea).

All candidates were subjected to similar living conditions during the entire hospitalization period and received the same hospital diet and followed a similar daily routine.

This study was approved by the Institutional Review Board of Beijing Huilongguan Hospital (Beijing Huilongguan Ethics Committee # 2019-43), and all participants provided written informed consent.

### Data Collection

All participants were interviewed on the day of admission. Patients' clinical symptoms were assessed by trained psychiatrists or psychologists using the 17-item HAM-D. The Pittsburgh Sleep Quality Index (PSQI) was assessed based on self-reported (subjective) sleep quality, including sleep duration, onset latency, sleep efficiency, sleep quality, sleep disturbance, sedative-hypnotic drugs, daily function, and total score over the past month (27). We used the Insomnia Severity Index (ISI) to evaluate the subjective perception of insomnia severity (28). As a seven-item and five-point Likert self-report questionnaire, the global score can range from 0 to 28 and is classified as follows: not clinically significant insomnia (0–7), subthreshold insomnia (8–14), moderate insomnia (15–21), and severe insomnia (22–28).

Fecal samples (≥1 g) collected within 2 days after admission or after the elution period were placed in a sample tube containing 2 ml of RNA stabilization solution (TinyGen, Bio-Tech, Shanghai, China) and stored at −80°C until DNA extraction.

Details on the methods for library preparation and 16S gene amplicon sequencing are provided in the Supplementary Methods. After sequence processing, according to different similarity levels, all sequences were divided into operational taxonomic units (OTUs), which were usually based on biological information statistical analysis at 97% similarity level.

### Determination of Bacterial Counts

DNA amplification was performed in the V4–V5 regions of the 16S rRNA gene and barcode sequences were added. Unique fusion primers were designed based on the general primers (515F 5′-GTGCCAGCMGCCGCGGTAA-3′, 926R 5′-CCGTCAATTCMTTTGAGTTT-3′). Sequencing was performed with Illumina 5′ (Illumina, San Diego, CA, USA) following the manufacturer's instructions (29). One unit Phusion DNA Polymerase (New England Biolabs, USA) was used to complete the initial PCR reactions. A DNA gel extraction kit (Axygen, USA) was used to purify the barcodes. PCR products and the FTC-3000™ real-time PCR (Funglyn, Shanghai) were used for quantification. Thermal cycling included an initial denaturation at 94°C for 2 min, followed by 25 cycles of denaturation at 94°C for 30 s, annealing at 56°C for 30 s, elongation at 72°C for 30 s, and a final extension at 72°C for 5 min. The PCR products from different samples were mixed at equal ratios. Eight PCR cycles were used to incorporate two unique barcodes to either of the ends of the amplicons. Finally, we used a DNA gel extraction kit (Axygen, USA) to purify the library and a 2 × 250-bp paired-end sequencing on the NovaSeq platform using NovaSeq 6000 SP 500 Cycle Reagent Kit (Illumina, USA) at TinyGen Bio-Tech (Shanghai) Co., Ltd.

Raw pyrosequencing reads were run through Trimmomatic (version 0.35) (30), and in order to remove low-quality base pairs, we used the parameters—SLIDINGWINDOW: 50:20, MINLEN: 50—while the FLASH program (version 1.2.11) was used to process default parameters. Low-quality contigs were removed based on the screen.seqs command using the following filtering parameters: maxambig = 0, minlength = 200, maxlength = 485, maxhomop = 8. A combination of software mothur (31) (version 1.33.3), UPARSE (usearch version v8.1.1756, http://drive5.com/uparse/) (32), and R (version 3.6.0) was used as quality filter. After sequence processing, according to different similarity levels, all sequences were divided into OTUs. Since OTUs are usually based on biological information statistical analysis at 97% similarity level, singleton OTUs were deleted using the UPARSE pipeline (http://drive5.com/uparse/).

### Bioinformatics and Statistical Analyses

For alpha diversity (Shannon, Simpson, and evenness indices), rarefaction curves were calculated using mothur and plotted by R. Phylogenetic beta diversity measures, weighted and unweighted UniFrac distance matrix were calculated using mothur and visualized with principal coordinate analysis (PCoA). Bray–Curtis and Jaccard metrics were calculated using the vegan package in R and visualized by R as UniFrac analysis. To compare within- and between-group similarity, analysis of similarity (ANOSIM) was performed with “vegan” package of R, based on (un) Weighted.unifrac distance. Canoco 5 RDA software was used to analyze the correlation between clinical indices and intestinal community variation.

Linear discriminant analysis effect size (LEfSe) analysis was used to identify taxa significantly enriched in the MDD and HC groups. The linear discriminant analysis (LDA) score was computed for taxa differentially abundant between the two groups. A taxon at *p* < 0.05 (Kruskal–Wallis test) and log10[LDA] ≥2.0 (or ≤ -2.0) were considered significant.

Demographic and clinical variables were compared between MDD and HC using the chi-square test for categorical variables, the independent-samples *t*-test for normal continuous variables, and the Mann–Whitney *U*-test for non-normal continuous variables. Taxa that have abundance >0.01% were reserved for analysis. The MDD group and HC were compared at the levels of phylum, class, order, family, genus, and species by the Wilcoxon signed-rank test. We performed partial correlations (adjusted for age, sex, and BMI) between bacterial counts and the total HAM-D-17 score. Moreover, partial correlation analysis (adjusted for age, sex, and BMI) was used to examine the correlations between bacterial counts and other variables such as total PSQI and ISI scores. Linear regression analyses were used to assess the most influential taxa on sleep quality in MDD at the genus and species levels after correcting for age, gender, BMI, and HAM-D scores. Differences were considered statistically significant when the two-tailed *p* < 0.05. False discovery rate (FDR, Benjamini–Hochberg) was used for perform multiple testing. *p* < 0.05 was considered significant. Analysis was performed using the Statistical Package for the Social Sciences version 25.0 (IBM Corp, Chicago, Illinois, USA).

## Results

### Demographic and Clinical Characteristics

Stool samples were collected from 36 patients with MDD and 45 healthy controls. Demographic and clinical characteristics were displayed in Table 1. There were no significant demographic differences between MDD and HC. However, the differences in HAM-D, ISI, and PSQI between the MDD and HC groups were significant. The mean age of onset was 31.39 ± 13.53 years, and illness duration was 5.36 ± 6.24 years. By thorough clinical tests, including blood and imaging examinations, participants had no local or systemic inflammation. We also collected the data of high-sensitivity C-reactive protein (hs-CRP) to exclude inflammation. They were all below the clinical threshold, and there was no significant difference between the two groups (Table 1).

**Table 1:** Demographic and clinical characteristics in MDD patients and HC.

**Parameter**	**MDD (*N* = 36)**	**HC (*N* = 45)**	**χ^**2**^/*t*/*Z***	***p*-value**
Age, years	36.81 ± 13.52	39.29 ± 11.44	−1.297	0.195
Gender, female/male	15/21	26/19	2.077	0.150
BMI, kg/m^2^	24.47 ± 4.16	23.94 ± 3.05	−0.656	0.513
Smoking, yes/no	6/30	5/40	0.526	0.468
Age of onset, years	31.39 ± 13.53	NA		
Illness duration, years	14.06 ± 3.04	NA		
HAM-D	5.36 ± 6.24	0.51 ± 1.01	−22.362	<0.001^***^
PSQI	23.06 ± 5.98	1.93 ± 1.29	−10.407	<0.001^***^
ISI	13.19 ± 7.79	0.40 ± 1.01	−9.755	<0.001^***^
hs-CRP	1.39 ± 1.34	1.03 ± 0.60	−0.57	0.954

*Data are presented as numbers or mean ± SD. MDD, major depressive disorder; HC, healthy controls (*

*** *p < 0.001). t stands for t score of independent-samples t-test. χ^2^ stands for χ^2^ score of the chi-square test. Z stands for Z score of the Mann–Whitney U-test*.

### Gut Microbiota Diversity Index Between MDD and HC

**Figure 1:**
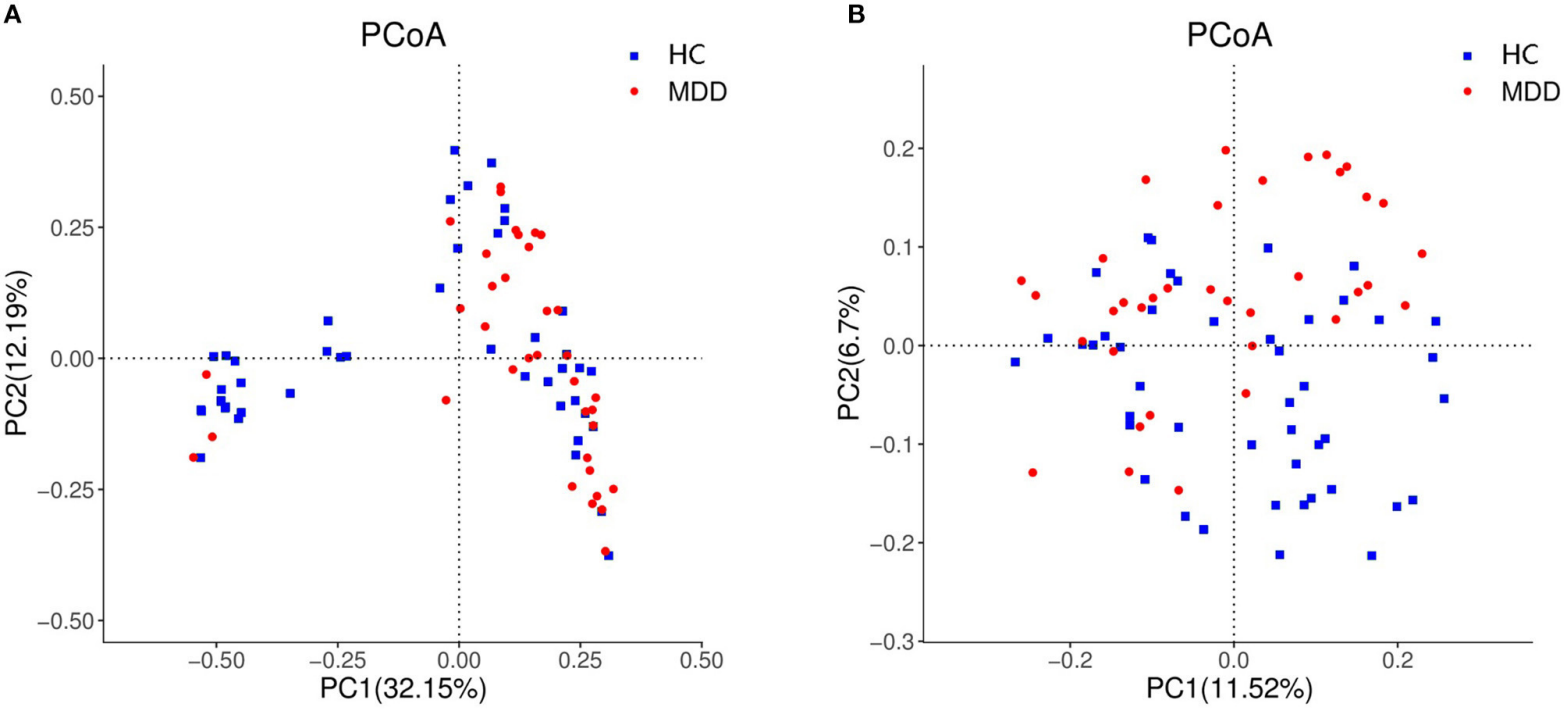
Beta diversity as a principal coordinate analysis (PCoA) plot based on Bray–Curtis dissimilarity **(A)** and Jaccard dissimilarity **(B)**.

**Figure 2:**
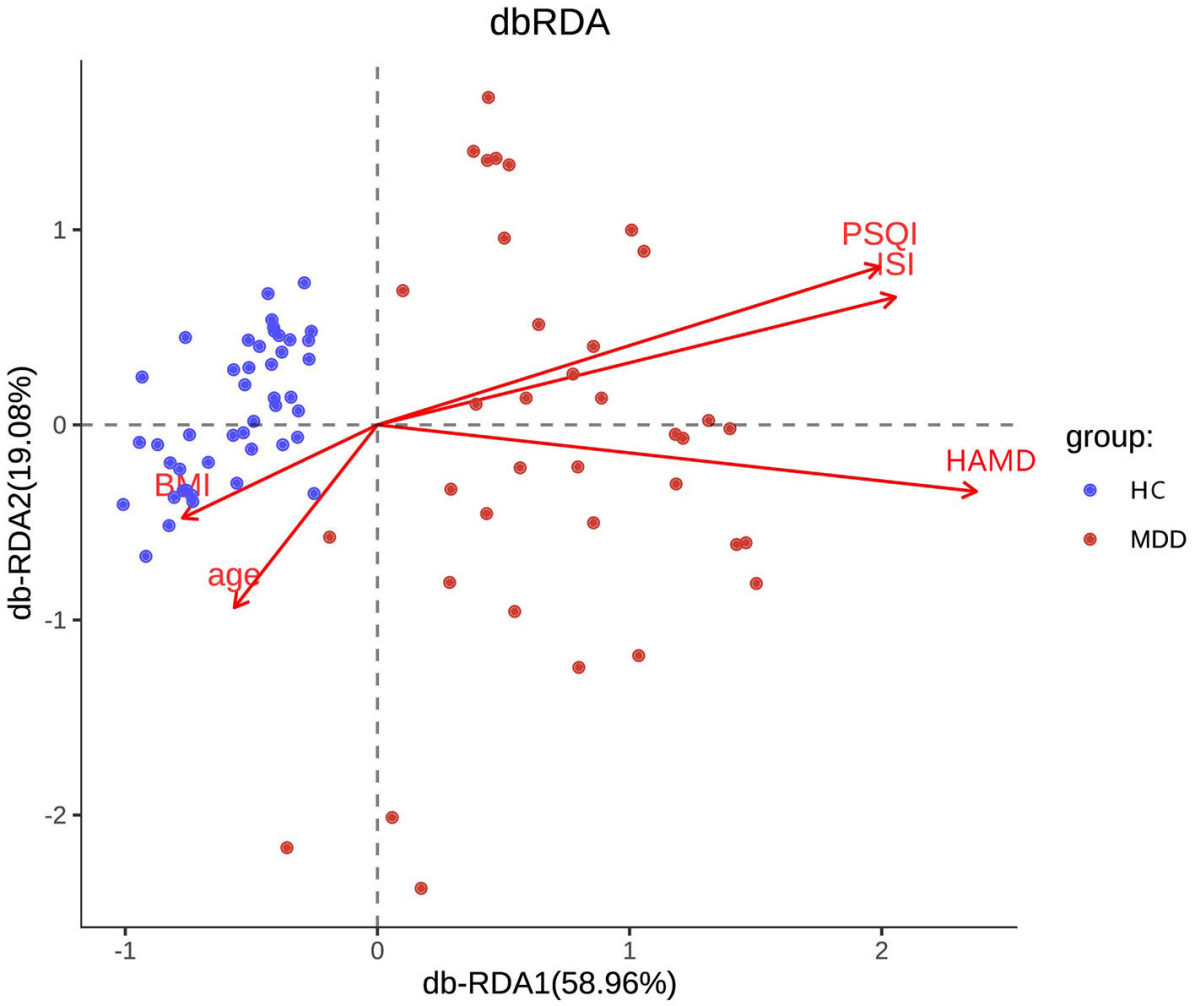
RDA two-way sequence diagram of intestinal bacterial community and its main environmental factors driven by variation.

**Figure 3:**
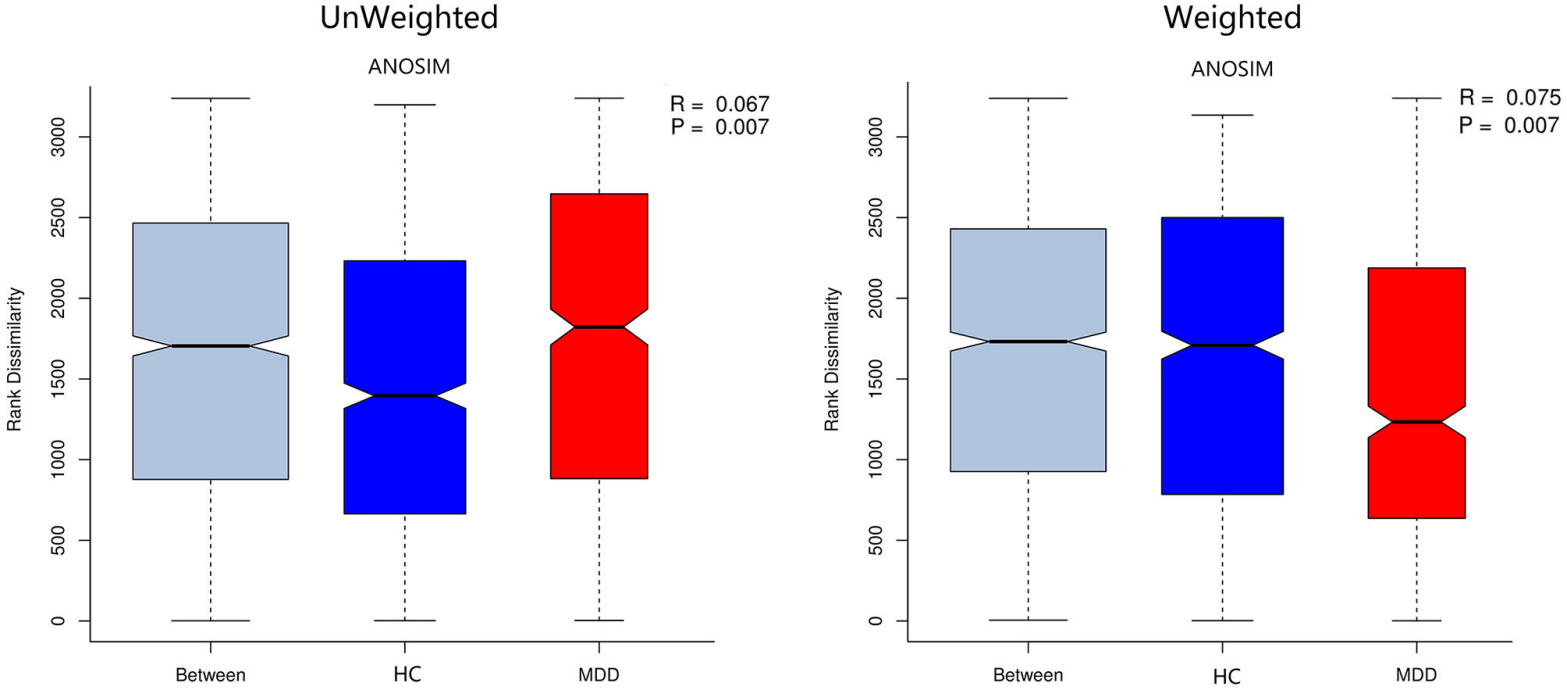
Unweighted and weighted analyses of similarities (ANOSIMs) based on the distance matrix of UniFrac differences of the fecal microbial communities in the MDD group and HC. *r* > 0 means the difference between groups is greater than that within groups.

Chao, ACE, Shannon, and Simpson indices were used to compare the alpha diversity between the MDD and HC groups. The Chao and ACE diversity indices reflect microbial species richness, while Shannon and Simpson diversity indices reflect community richness and evenness. No difference in alpha diversity between patients with MDD and HC was observed (*p* > 0.05). Differences in community structure dispersion between the two groups were measured using Bray–Curtis analysis (Figure 1A), and community membership dispersion was assessed by Jaccard dissimilarity (Figure 1B). We used Canoco 5 RDA software to analyze the correlation between clinical indices and intestinal community variation. The relationship between the distribution of microflora and clinical indices is shown in Figure 2. PSQI, HAM-D, and ISI played important roles on the distribution of microflora in MDD patients, while BMI and age played important roles on HC. Among them, HAM-D has the most significant effect on community variation (*p* < 0.05) (Supplementary Table 5). Furthermore, unweighted and weighted ANOSIMs revealed significant differences between the two groups. Beta diversity was measured based on the unweighted (*r* = 0.067, *p* = 0.007) and the weighted (*r* = 0.075, *p* = 0.007) UniFrac distance matrix of the differences between groups, as shown in Figure 3, suggesting dissimilar microbiota composition.

### Composition of Microbial Communities Between MDD and HC

**Figure 4:**
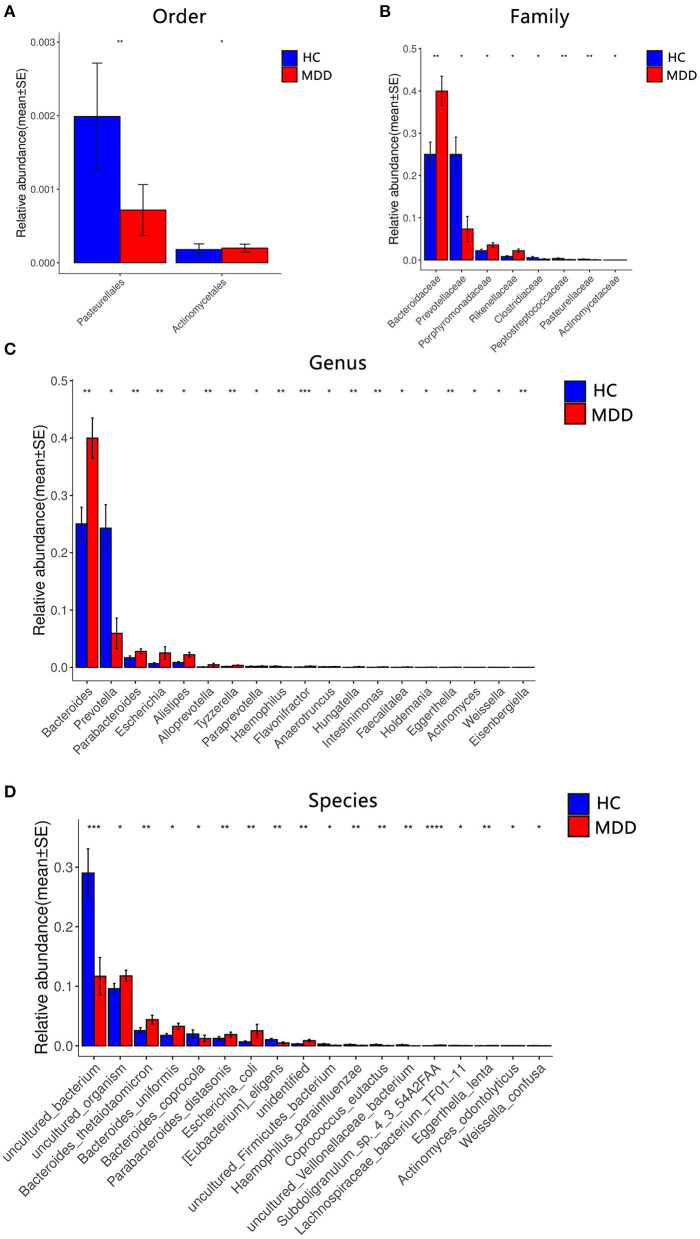
Comparison of the microbial abundance among the MDD group and HC. Dominant bacteria with relative abundances >0.01%. After exclusion, the Wilcoxon signed-rank test was applied to identify the differentially abundant orders **(A)**, families **(B)**, genera **(C)**, and species **(D)**. Among these, the highest means of the phylogenetic abundance in the enriched cohort were drawn as bar plots (*p < 0.05, **p < 0.01, ***p < 0.001, ****p < 0.0001).

We obtained 3,565,920 quality-filtered read pairs from 81 study participants (36 MDD patients and 45 HC), with an average of 44,024 read pairs per sample. Gut bacterial communities at the phylum, family, and genus levels detected in MDD and HC subjects are shown in Figure 4. In order to explore the differences among groups, all OTUs with ≥0.01% fractional representation in either of the groups were considered. As expected, *Bacteroidetes* and *Firmicutes* accounted for about 93% of all bacteria and were the two most common dominant taxa among the two groups. At the phylum and class levels, there were no significant differences between groups (*p* > 0.05).

A significant difference (Wilcoxon signed-rank test, *p* < 0.05) between the two groups affected two orders: *Pasteurellales* and *Actinomycetae*. At the family level, eight families showed significant differences between the two groups: the top four being *Bacteroidaceae, Prevotellaceae, Porphyromonadaceae*, and *Rikenellaceae*. At the genus level, 19 genera showed significant differences between the two groups; the top four were *Bacteroides, Prevotella, Parabacteroides*, and *Escherichia*. At the species level, 18 species showed significant differences between the two groups; the top four were *uncultured_bacterium, uncultured_organism, Bacteroides_thetaiotaomicron*, and *Bacteroides_uniformis*. After the strict FDR correction, at the genus level, 10 genera showed significant differences between the two groups; the top four were *Flavonifractora, Alloprevotella, Parabacteroides*, and *Hungatella*. At the species level, four species showed significant differences between the two groups; they were *Subdoligranulum_sp._4_3_54A2FAA, uncultured_bacterium, Eggerthella_lenta*, and *uncultured_Veillonellaceae_bacterium*. No significant differences were identified at the family and order levels. The abundance of these microbiota targets is presented in Figure 4, Table 2, and Supplementary Table 1.

**Table 2:** Taxa abundances in MDD and HC.

**Parameter**	**MDD**	**HC**	***p*-value**	***p* adj**.
	**(*N* = 36)**	**(*N* = 45)**		
**Order**				
*Pasteurellales*	0.07%	0.20%	0.005^**^	0.082
*Actinomycetales*	0.02%	0.02%	0.015^*^	0.131
**Family**				
*Bacteroidaceae*	40.00%	25.03%	0.003^**^	0.099
*Prevotellaceae*	7.36%	25.02%	0.028^*^	0.119
*Porphyromonadaceae*	3.59%	2.22%	0.017^*^	0.086
*Rikenellaceae*	2.22%	0.86%	0.046^*^	0.172
*Clostridiaceae*	0.22%	0.57%	0.015^*^	0.089
*Peptostreptococcaceae*	0.11%	0.39%	0.009^**^	0.086
*Pasteurellaceae*	0.07%	0.20%	0.005^**^	0.068
*Actinomycetaceae*	0.02%	0.02%	0.015^*^	0.109
**Genus**				
*Bacteroides*	40.00%	25.03%	0.003^**^	0.045^*^
*Prevotella*	5.95%	24.29%	0.015^*^	0.079
*Parabacteroides*	2.79%	1.68%	0.005^**^	0.043^*^
*Escherichia*	2.53%	0.67%	0.009^**^	0.054
*Alistipes*	2.22%	0.86%	0.044^*^	0.155
*Alloprevotella*	0.47%	0.08%	0.002^**^	0.042^*^
*Tyzzerella*	0.35%	0.16%	0.007^**^	0.049^*^
*Paraprevotella*	0.20%	0.17%	0.028^*^	0.110
*Haemophilus*	0.07%	0.20%	0.006^**^	0.046
*Flavonifractor*	0.21%	0.07%	<0.001^**^	0.010^*^
*Anaerotruncus*	0.14%	0.11%	0.033^*^	0.123
*Hungatella*	0.10%	0.01%	0.005^**^	0.044^*^
*Intestinimonas*	0.08%	0.01%	0.003^**^	0.046^*^
*Faecalitalea*	0.06%	0.02%	0.027^*^	0.113
*Holdemania*	0.04%	0.01%	0.017^*^	0.082
*Eggerthella*	0.04%	0.01%	0.002^**^	0.059
*Actinomyces*	0.02%	0.02%	0.015^*^	0.081
*Weissella*	0.00%	0.02%	0.024^*^	0.109
*Eisenbergiella*	0.01%	0.01%	0.004^**^	0.048^*^
**Species**				
*uncultured_bacterium*	11.68%	29.01%	<0.001^***^	0.027^*^
*uncultured_organism*	11.75%	9.59%	0.049^*^	0.207
*Bacteroides_thetaiotaomicron*	4.39%	2.56%	0.006^**^	0.055
*Bacteroides_uniformis*	3.28%	1.74%	0.026^*^	0.134
*Bacteroides_coprocola*	1.23%	1.98%	0.028^*^	0.128
*Parabacteroides_distasonis*	1.89%	1.25%	0.010^*^	0.060
*Escherichia_coli*	2.53%	0.67%	0.009^**^	0.060
*Unidentified*	0.88%	0.32%	0.008^**^	0.061
*uncultured_Firmicutes_bacterium*	0.09%	0.30%	0.026^*^	0.128
*Haemophilus_parainfluenzae*	0.07%	0.20%	0.006^**^	0.060
*Coprococcus_eutactus*	0.03%	0.19%	0.005^**^	0.054
*uncultured_Veillonellaceae_bacterium*	0.00%	0.16%	0.002^**^	0.040^*^
*Subdoligranulum_sp._4_3_54A2FAA*	0.10%	0.01%	<0.001^***^	<0.001^***^
*Lachnospiraceae_bacterium_TF01–11*	0.03%	0.05%	0.040^*^	0.017^*^
*Eggerthella_lenta*	0.04%	0.01%	0.002^**^	0.039^*^
*Actinomyces_odontolyticus*	0.02%	0.02%	0.016^*^	0.092
*Weissella_confusa*	0.00%	0.02%	0.024^*^	0.134
*[Eubacterium]_eligens*	0.48%	1.04%	0.005^**^	0.057

*Data are presented as mean relative abundance of gut microbiome in MDD groups and HC (*

* *p < 0.05*,

** *p < 0.01*,

*** *p < 0.001). p adj. stands for p value after FDR correction*.

**Figure 5:**
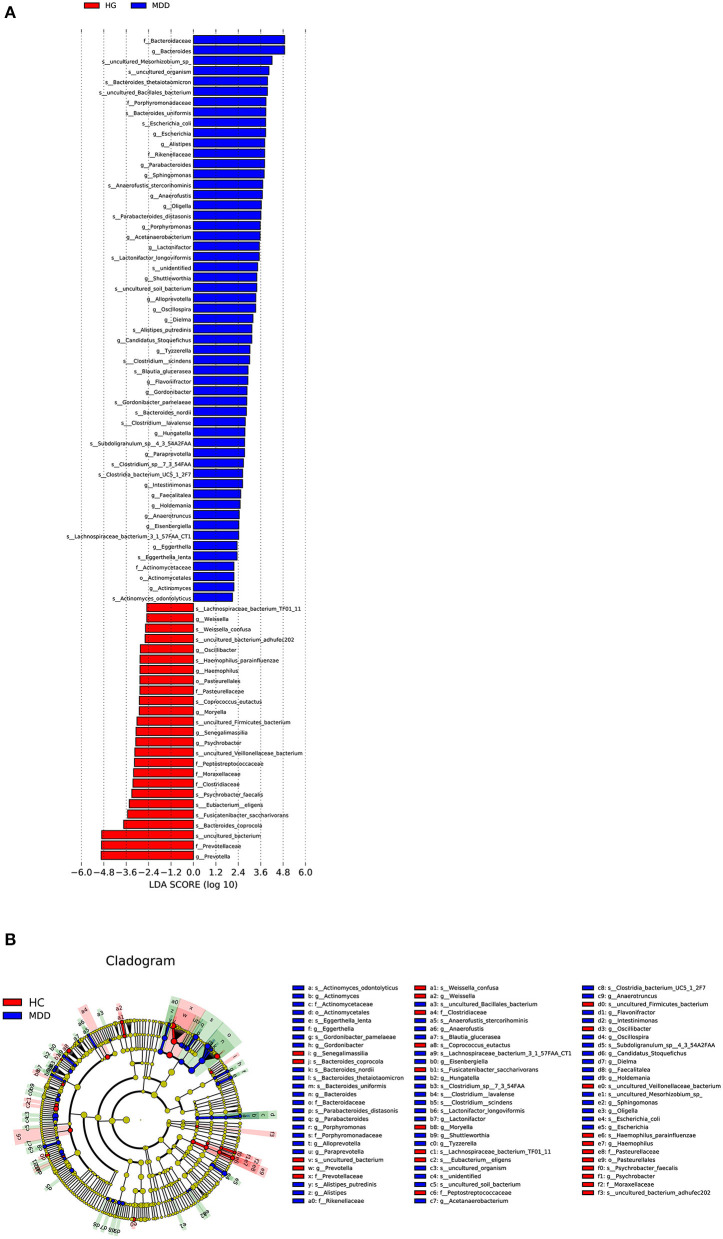
**(A)** Taxonomic biomarkers found by LEfSe in HC (blue) and MDD (red). Only taxa with *p* < 0.05 and LDA score (log10) are shown. **(B)** Cladogram indicating the phylogenetic relatedness of the discriminant taxa.

Based on the LDA score, we found that *Bacteroidaceae, Bacteroides*, and *uncultured_Mesorhizobium_sp*. were associated with healthy controls and *uncultured_bacterium, Prevotellaceae*, and *Prevotella* with the MDD group (Figure 5).

### Correlations of Microbiota Abundance With Severity of Depression and Sleep Quality in MDD

After controlling for potential confounders (age, sex, BMI), the results of partial correlation analysis indicated no correlation between alpha diversity and ISI and HAM-D scores, while a moderate correlation was observed between Chao and PSQI scores (*r* = −0.345, *p* = 0.049). At the genus level, *Dorea* (*r* = 0.355, *p* = 0.042), *Butyricicoccus* (*r* = 0.364, *p* = 0.037), and *Peptococcu*s (*r* = 0.403, *p* = 0.020) showed moderate correlations with the HAM-D score, while *Blautia* (*r* = 0.406, *p* = 0.019), *Coprococcus* (*r* = 0.508, *p* = 0.003), *Dorea* (*r* = 0.399, *p* = 0.022), and *Intestinibacter* (*r* = −0.559, *p* = 0.001) showed moderate to strong correlations with the PSQI score, and *Intestinibacter* (*r* = −0.489, *p* = 0.004) a moderate one with the ISI score. In addition, the species *Bacteroides_stercoris* (*r* = −0.368, *p* = 0.035), *Bacteroides_uniformis* (*r* = 0.424, *p* = 0.014), and *Parasutterella_secunda* (*r* = 0.358, *p* = 0.041) showed moderate correlations with HAM-D score; *Blautia_obeum* (*r* = −0.392, *p* = 0.024), *Streptococcus_salivarius_subsp._salivarius* (*r* = −0.352, *p* = 0.045), *Coprococcus_comes* (*r* = 0.408, *p* = 0.018), *Blautia_sp*. (*r* = −0.411, *p* = 0.018), *butyrate_producing_bacterium_L250* (*r* = −0.357, *p* = 0.041), *Dorea_formicigenerans* (*r* = −0.419, *p* = 0.015), and *uncultured_Coprococcus_sp*. (*r* = −0.345, *p* = 0.049) show moderate correlation with PSQI score; and *uncultured_Clostridiales_bacterium* (*r* = 0.357, *p* = 0.042) and *Blautia_obeum* (*r* = −0.350, *p* = 0.046) show moderate correlation with ISI. Correlations with HAM-D, PSQI, and ISI scores are shown in Table 3 and Supplementary Table 2. Notably, *Dorea* commonly correlated with HAM-D and PSQI scores in patients with MDD. After FDR correction, at the genera level, *Intestinibacter* with PSQI was still significant. At the species level, no correlation was shown.

**Table 3:** Partial correlations between taxa and HAM-D, PSQI, and ISI scores in MDD.

**Taxa**	**HAM-D**			**PSQI**			**ISI**		
	**MDD**			**MDD**			**MDD**		
	***r***	***p*-value**	***p* adj**.	***r***	***p*-value**	***p* adj**.	***r***	***p*-value**	***p* adj**.
**Genus**									
*Blautia*	0.039	0.830	0.99	−0.406	0.019^*^	0.431	−0.226	0.207	0.999
*Coprococcus*	−0.236	0.186	0.99	−0.508	0.003^**^	0.101	−0.242	0.174	0.999
*Dorea*	−0.355	0.042^*^	0.99	−0.399	0.022^*^	0.374	−0.247	0.166	0.999
*Butyricicoccus*	0.364	0.037^*^	0.99	−0.069	0.705	0.888	0.065	0.720	0.999
*Intestinibacter*	−0.215	0.229	0.99	−0.559	0.001^**^	0.048^*^	−0.489	0.004^**^	0.272
*Peptococcus*	0.403	0.020^*^	0.99	−0.195	0.277	0.897	0.038	0.835	0.999
**Species**									
*Bacteroides_stercoris*	−0.368	0.035^*^	0.999	−0.007	0.97	0.994	0.072	0.689	0.833
*Bacteroides_uniformis*	0.424	0.014^*^	0.999	−0.025	0.89	0.994	0.231	0.196	0.838
*uncultured_Clostridiales_bacterium*	−0.117	0.516	0.999	0.124	0.491	0.815	0.357	0.042^*^	0.84
*Blautia_obeum*	−0.072	0.692	0.999	−0.392	0.024^*^	0.528	−0.35	0.046^*^	0.855
*Streptococcus_salivarius_subsp._salivarius*	−0.192	0.284	0.999	−0.352	0.045^*^	0.566	−0.164	0.362	0.857
*Coprococcus_comes*	−0.178	0.323	0.999	−0.408	0.018^*^	0.792	−0.061	0.736	0.863
*Blautia_sp*.	0.061	0.735	0.999	−0.411	0.018^*^	0.528	−0.098	0.588	0.864
*butyrate-producing_bacterium_L2-50*	−0.249	0.162	0.999	−0.357	0.041^*^	0.601	−0.204	0.255	0.878
*Dorea_formicigenerans*	−0.285	0.108	0.950	−0.419	0.015^*^	0.994	−0.197	0.273	0.881
*uncultured_Coprococcus_sp*.	0.049	0.787	0.999	−0.345	0.049^*^	0.539	−0.192	0.284	0.881
*Parasutterella_secunda*	0.358	0.041^*^	0.999	−0.052	0.775	0.922	0.229	0.200	0.882

*Partial correlation analysis between taxa and HAM-D, PSQI, and ISI in MDD or controls, after controlling for age, sex, and BMI. HAM-D, Hamilton Depression Rating Scale; PSQI, Pittsburgh Sleep Quality Index; ISI, Insomnia Severity Index (*

* *p < 0.05*,

** *p < 0.01). p adj. stands for p-value after FDR correction*.

In HC, after controlling for potential confounders (age, sex, BMI), the results of partial correlation analysis are shown in Supplementary Table 2. At the genus level, *Haemophilus* (*r* = 0.517, *p* < 0.001) showed strong correlations with the HAM-D score, *Acidaminococcus* (*r* = −0.345, *p* = 0.025) showed moderate correlations with the PSQI score, and *Haemophilus* (*r* = 0.753, *p* < 0.001) had a strong correlation with the ISI score. In addition, the species *Haemophilus_parainfluenzae* (*r* = 0.517, *p* < 0.001) showed strong correlations with HAM-D score. *Haemophilus_parainfluenzae* (*r* = 0.752, *p* < 0.001) and *Clostridium_paraputrificum* (*r* = 0.670, *p* < 0.001) showed strong correlations with ISI. After FDR correction, at the genera level, the correlations between *Haemophilus* and HAM-D and ISI were still significant. At the species level, *Haemophilus_parainfluenzae* with HAM-D, *Haemophilus_parainfluenzae*, and *Clostridium_paraputrificum* with ISI were still significant.

When adding severity of depression to potential confounding factors, the most relevant taxa with sleep quality in MDD at the genus levels were *Coprococcus* (β = −0.322, *p* = 0.021) and *Intestinibacter* (β = −0.455, *p* = 0.006), and the results are shown in Table 4.

**Table 4:** Linear regression analysis results for PSQI at the genus level.

	**Unstandardized**		**Standardized**	***t***	***p*-value**
	**Beta**	**SE**	**Beta**		
HAM−D score	0.160	0.126	0.199	1.269	0.214
Gender	−1.381	1.296	−0.143	−1.066	0.295
Age	0.095	0.052	0.266	1.832	0.077
BMI	−0.135	0.169	−0.117	−0.799	0.431
*Intestinibacter*	−10505.272	3521.040	−0.455	−2.984	0.006^**^
*Coprococcus*	−281.100	115.270	−0.322	−2.439	0.021^*^

*Linear regression analyses were used to assess the most influential taxa on sleep quality in MDD at the genus and species levels after correcting for age, gender, BMI, and HAM-D scores. PSQI, Pittsburgh Sleep Quality Index (*

* *p < 0.05*,

** *p < 0.01)*.

### Differences of MDD Patients With or Without Sleep Disorder

**Figure 6:**
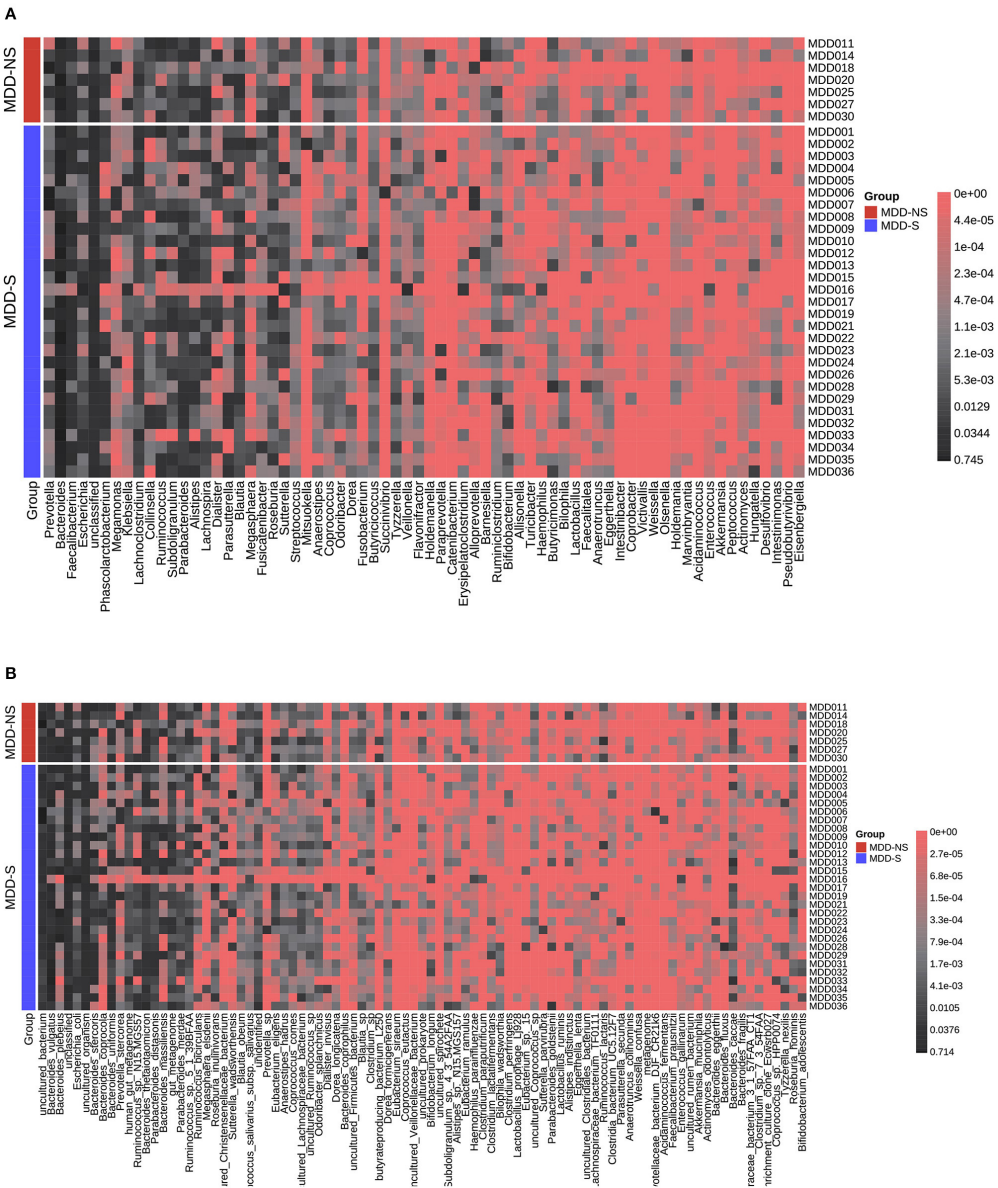
Relative abundance distribution for MDD-S and MDD-NS at the genus level **(A)** and species level **(B)**. MDD-S stands for MDD patients with sleep disorder (PSQI > 5), and MDD-NS stands for MDD patients without sleep disorder (PSQI ≤ 5).

We divided the MDD patients into two groups according PSQI scores (>5). Figures 6A,B show the relative abundance of all the patients at the genus level and species level, respectively. A significant difference was shown between the two groups (Supplementary Table 3). At the genus level, *Streptococcus, Dorea, Barnesiella*, and *Intestinibacter* decreased in MDD patients with sleep disorder, while *Coprococcus* increased. At the species level, *Blautia_obeum, Streptococcus_salivarius_subsp*._*salivarius, Dorea_formicigenerans, uncultured_Coprococcus_sp*., and *Ruminococcus_lactaris* decreased in MDD patients with sleep disorder, and *Clostridium_sp*. increased. After FDR correction, no significant difference was found between the two groups.

## Discussion

Our study demonstrated significant gut flora differences between MDD and HC and found partially similar results to previous studies, as well as differences. Notably, we explore the composition of the microbiota of patients with MDD in relation to sleep quality and the severity of insomnia. We considered medication factors by limiting drug natural washout period, and we enrolled patients without any treatment >1 week and without long-acting antipsychotics >6 months.

In our study, some of the microbiota targets at the order, family, genus, and species levels indicated significant differences between MDD and HC. At the family level, a higher abundance of *Actinomycineae* and *Porphyromonadaceae* and a lower abundance of *Prevotellaceae* were observed in MDD (11, 33). At the genus level, higher *Bacteroides, Parabacteroides*, and *Alistipes* and lower *Prevotella* and *Eggerthella* were observed in MDD (11, 34–36). These results are consistent with previous reports. Transplanting the fecal flora from depressive patients to mice showed similar behaviors and microflora phenotype in those mice as the donor patients, indicating that the gut microbiome could cause depressive symptoms by affecting the metabolism (37). Growing research supports the effect of microbiota on brain networks and the regulation of negative affect. *Alistipes* can produce indole to influence tryptophan metabolism, which is vital for emotion regulation (38). However, the lower abundance of *Peptostreptococcaceaea* and *Rikenellaceae* in MDD was not found in our study. *Rikenellaceae* is a butyrate producer that can attenuate inflammation levels in order to improve emotion (39). *Peptostreptococcaceaea* belongs to *Firmicutes* which can affect glucose metabolism to mediate inflammation levels (40). A neuroimaging study found that patients with MDD had higher concentrations of *Bacteroides* and lower *Prevotella* and showed differences of brain structure and function associated with emotion (41). Age, BMI, gender, nations, diet, region, diagnostic criteria, mediation, lifestyles, sample size, and subclinical symptoms often disturb the reproducibility and accuracy of results (11, 42). In our study, age, BMI, and gender were similar between the two groups, and a mediation factor was also considered. Interestingly, our study found that *Dorea, Butyricicoccus*, and *Peptococcus* were associated with HAM-D scores measuring the severity of depression, which has never been reported before (9, 26, 43, 44).

Another contribution of our study was to further clarify the correlation of gut microbiota and concomitant sleep symptoms. We clustered the gut bacterial distributions based on existing similarity. PSQI and ISI represent different aspects of sleep, unlike ISI, sleep latency, sleep duration, and sleep efficiency components based on free-text numerical responses, while ISI concentrated on perceived feeling (45). At the genus level, *Blautia, Coprococcus, Dorea*, and *Intestinibacter* were negatively correlated with PSQI, with *Intestinibacter* being simultaneously negatively correlated with PSQI and ISI. Moreover, in our study, after controlling for the HAM-D score, *Coprococcus* and *Intestinibacter* were associated with sleep quality, independent of the severity of depression. However, contrary to our findings, a recent study showed that higher sleep quality was associated with a high proportion of bacteria from the *Verrucomicrobia* and *Lentisphaerae* phyla (46). Recent reviews have summarized the potential mechanism of sleep and MBGA: immunoregulatory pathway (cytokines), neuroendocrine pathway (HPA axis, CNS, neurotransmitters), vagus nerve pathway, and gut microbial metabolite pathway (SCFAs) (20, 47). Butyrate producers (*Blautia, Coprococcus*) may influence sleep quality because butyrate may potentially serve as a sleep-inducing signal molecule to enhance sleep (48), and the results were consistent with those of previous studies (49). Depressive patients were observed to exhibit similar phenomena, indicating that the two gut bacteria were more relevant to sleep rather than depressive symptoms. Depression and sleep are both affected by circadian activity, and no single hypothesis can explain the complex mechanisms of the comorbidity of depression and insomnia (20). *Dorea* was observed to decrease with HAM-D and PSQI. Thus, altered gut microbiota composition may correlate not only with an increased MDD severity but also with lower sleep quality. Indeed, Huang et al. found that *Dorea* decreased in depressive patients, although no previous study has reported its link with sleep (50). Since *Dorea* is known for fermenting polysaccharides into SCFAs (51) and SCFAs, including butyrate and acetate, play important roles in clock gene expression, which is closely related to circadian rhythm and sleep quality, it is possible that *Dorea* mediates sleep deficits in MDD (52). However, it is not clear why *Intestinibacter* is associated with sleep, whether *via* immune inflammatory mechanisms or carbohydrate metabolism (53–55).

In our study, the correlation of gut microbiome and clinical symptoms in patients and in cases and controls was different. Previous studies reported the correlation between gut microbiome and sleep quality in animals or in healthy controls. *Blautia* and *Ruminococcus* were reported to have a negative correlation with PSQI score, and *Prevotella* was positively correlated with PSQI score in young healthy individuals (22). A recent study reported that probiotics could improve sleep quality and has a role in anti-inflammatory mechanism (47). Our study identified that *Acidaminococcus* was associated with better sleep quality, and it also identified the heterogeneity of microbiome in MDD and HC. Different gut flora were associated with different metabolites, metabolic pathways, and inflammatory pathways. Sleep and circadian rhythms could influence microbiome composition by inflammation and breakdown of the epithelial barrier (56). The inflammatory reaction mechanism is induced by the microbiome and then triggers the CNS and aggravates insomnia and depression (57).

As for alpha diversity, our results are consistent with the latest meta-analysis, which found no difference in alpha diversity between patients with MDD and HC (58). History studies showed ambiguous results of beta diversity, including increasing and no difference due to individual heterogeneity and using different measuring instruments and analysis software (35, 36, 59). Our results reported different microbiota compositions between the two groups, similar to Chung et al. (11). In the future, more extensive sampling is needed to verify the results. Most scholars believe that the higher the microbiome diversity, the better the health (60). Zhang et al. reported no overt changes in microbiome richness or composition after sleep restriction (21). However, another study indicated that alpha diversity was positively associated with sleep efficiency and total sleep time and negatively associated with sleep fragmentation (49). The marginal significance in community richness observed in our study may be limited by the small sample size, while the short-term loss of sleep may not affect the gut microbiome diversity (61). It is worth noting that RDA analysis pointed out that sleep quality, severity of insomnia, and depression were main environmental factors, which drove the variation of microbiome in MDD patients not in HC. Therefore, in MDD patients, clinical symptoms were significantly related to the changes of microflora.

Considering our results, some limitations were noted. First, part of our results did not pass strict FDR correction (*p* adj. value should be <0.05), and we believe the negative results failing multiple testing would give a systematic view regarding our study. Also, the small sample size with limited power makes it impossible to identify more related factors. Further studies are needed to enlarge the sample size. Second, a cross-sectional study cannot prove causality; therefore, a longitudinal study is warranted. Although we controlled medication and diet after admission to minimize confounding, long-term dietary and medication effects should be considered. Our healthy controls consisted of hospital workers (*N* = 14) and patients' families or friends with no consanguinity (*N* = 31). They were all from Beijing, with similar geographical location, and in relatively consistent dietary habits. As a previous study pointed out that there were differences in the microbiome of hospital workers and non-hospital workers (62), we further compared the difference between healthy hospital workers (HCW) and non-hospital workers (non-HCW). There was no significant difference between them in our study (Supplementary Table 4). Recruiting patients focusing on the same area, applying the dietary questionnaire, and collecting the patients' medication history will help increase the reliability of results. Thirdly, we did not measure the stool moisture in our research, because the stools were immediately placed in a sample tube containing 2 ml of RNA stabilization solution. Further study should consider the impact of stool consistency. Additionally, as a common sequencing method, we used 16S RNA sequences, despite new methods such as shotgun metagenomics often reflecting more reliability and repeatability, but at a higher cost. Lastly, functional microbiota analysis, immunological status, gut barrier integrity, and metabolomics should also be integrated in future studies.

## Conclusions

The microbiomes of patients with major depressive disorder in China were found to be significantly different from those of healthy controls. In summary, 48 microbiota targets were associated with MDD, 6 with severity of depression, 11 with sleep quality, and 3 with insomnia severity. At the genus level, *Dorea* was simultaneously related to depression and sleep quality, and *Intestinibacter* was more closely associated with sleep-related problems. The most interesting finding was that the presence of *Coprococcus* and *Intestinibacter* was associated with sleep quality independent of the severity of depression. We identified several specific taxa related to sleep health, which suggests that the microbiome may be related to both sleep and severity of illness at the same time. Overall, our findings are consistent with previous microbiome studies on depression and constitute a preliminary exploration of the role of dysbacteriosis in sleep disorders in MDD patients.

## Data Availability Statement

The datasets presented in this study can be found in online repositories. The names of the repository/repositories and accession number(s) can be found at: https://www.ncbi.nlm.nih.gov/, PRJNA687871.

## Ethics Statement

The studies involving human participants were reviewed and approved by the Institutional review board of Beijing Huilongguan Hospital (# 2019–43). The patients/participants provided their written informed consent to participate in this study.

## Author Contributions

QZ conducted data collection and analysis and drafted and revised the manuscript. FY and ZW designed the experiments. YY, WZ, and TM collected the data. HA proofread the manuscript. All authors contributed to the article and approved the submitted version.

## Conflict of Interest

The authors declare that the research was conducted in the absence of any commercial or financial relationships that could be construed as a potential conflict of interest.
